# High serum uric acid levels are protective against cognitive impairment in amyotrophic lateral sclerosis

**DOI:** 10.1007/s00415-023-12056-8

**Published:** 2023-10-25

**Authors:** Barbara Iazzolino, Maurizio Grassano, Cristina Moglia, Antonio Canosa, Umberto Manera, Rosario Vasta, Sara Cabras, Stefano Callegaro, Enrico Matteoni, Francesca Di Pede, Francesca Palumbo, Gabriele Mora, Andrea Calvo, Adriano Chiò

**Affiliations:** 1 Department of Neuroscience “Rita Levi Montalcini”, ALS Center, University of Turin, Via Cherasco 15, 10126 Turin, Italy; 2 Neurology I, Azienda Ospedaliero-Universitaria Città della Salute e della Scienza di Torino, Turin, Italy; 3 Institute of Cognitive Science and Technologies, National Research Council, Rome, Italy; 4 International School of Advanced Studies, University of Camerino, Camerino, Italy

**Keywords:** Amyotrophic lateral sclerosis, Cognition, Frontotemporal dementia, Uric acid, Phenotype

## Abstract

**Background:**

Uric acid (UA) has emerged as a factor that can modify cognitive function both in the general population and in people with neurodegenerative disorders. Since very few data are available concerning amyotrophic lateral sclerosis (ALS), we explored the correlation of UA levels and cognitive impairment in a large cohort of ALS patients.

**Methods:**

We enrolled ALS patients consecutively seen at the Turin ALS expert center in the 2007–2018 period who underwent both cognitive/behavioral and UA evaluation at diagnosis. Patients were classified in 5 categories: normal cognition (ALS-CN), isolated cognitive impairment (ALSci), isolated behavioural impairment (ALSbi), cognitive and behavioural impairment (ALScbi), frontotemporal dementia (ALS-FTD). For this study, ALSci, ALSbi and ALScbi were merged as ALS with intermediate cognitive impairment (ALS-INT).

**Results:**

Out of the 841 ALS patients, 422 had ALS-CN, 271 ALS-INT and 148 ALS-FTD. The mean values of UA were significantly different among the cognitive subgroups of patients, with the lowest values in the ALS-FTD (ALS-CN, 288.5 ± 78.0 (μmol/L; ALS-INT, 289.7 ± 75.5 μmol/L; ALS-FTD, 271.8 ± 74.9 μmol/L; *p* = 0.046). The frequency of ALS-FTD was significantly higher in the 1st tertile of UA. Lower UA levels were independently associated with FTD (OR 1.32, 95% c.i. 1.01–1.43; *p* = 0.038) in binary logistic regression.

**Conclusions:**

We found that in ALS lower UA serum levels are correlated with reduced frequency of co-morbid FTD. Patients with intermediate cognitive impairment showed UA levels similar to ALS-CN but higher than ALS-FTD, implying that higher UA levels can prevent or delay cognitive function deterioration.

**Supplementary Information:**

The online version contains supplementary material available at 10.1007/s00415-023-12056-8.

## Introduction

Amyotrophic lateral sclerosis (ALS) is a complex neurodegenerative disorder encompassing motor and cognitive disturbances [1]. ALS clinical heterogeneity, and in particular the presence of cognitive impairment, has profound negative effects on the course of the disease and impacts both the planning of the management and the design and interpretation of clinical trials. The causes of ALS phenotypical heterogeneity are still incompletely understood and may include the different pathogenetic mechanisms involved in the disease, i.e., genetic, epigenetic, and environmental factors [1, 2]. A better understanding of these mechanisms and their interactions will help to improve the treatment of ALS patients.

Uric acid (UA) has emerged among the factors that can modify cognitive function both in the general population and in people with neurodegenerative diseases such as Parkinson’s disease (PD) [3]. However, despite several biological mechanisms that have been proposed for this relationship, reverse causality has not been definitely excluded. The aim of this study is to explore the correlation of serum UA levels and cognitive impairment in a large cohort of ALS patients.

## Methods

The study population includes ALS patients who were consecutively seen at the Turin ALS expert center in the 2007–2018 period, and who underwent both cognitive/behavioral testing and serum UA evaluation dosage. A comprehensive battery of cognitive tests was administered within 2 months of diagnosis. Fasting serum UA was assessed as part of the diagnostic work-up and is reported as μmol/L. Patients were diagnosed as definite, probable, probable laboratory-supported and possible ALS according to El Escorial revised criteria [4]. At the time of cognitive testing the ALS Functional Rating Scale–revised (ALSFRS-R) score was collected. Patients with a history of disorders which may potentially affect cognition (i.e., major stroke, severe head injuries, mental retardation), alcohol or drug dependence, severe mental illness, or use of high-dose psychoactive medications were tested but not included in data analysis. Patients who were not in Italian native language were assessed only through an unstructured interview and therefore were excluded from the analysis.

### Neuropsychological assessment

Patients and controls underwent a battery of neuropsychological tests encompassing executive function, verbal and visual memory, attention and working memory, visuospatial function, language, social cognition, and behavior selected according to the Diagnostic Criteria for the Behavioural variant of Frontotemporal Dementia [5], and ALS-FTD Consensus Criteria (ALS-FTDC) [6]. The list of tests included in the battery are reported in the Supplemental methods. According to ALS-FTDC patients were classified in 5 categories: patients with normal cognition (ALS-CN), patients with isolated cognitive impairment (ALSci), patients with isolated behavioural impairment (ALSbi), patients with both cognitive and behavioural impairment (ALScbi) and patients with frontotemporal dementia (ALS-FTD) [6]. For this study, ALSci, ALSbi and ALScbi were merged as ALS with intermediate cognitive impairment (ALS-INT).

### Statistical methods

Binary logistic regression analysis (backward) was performed, adjusting for sex, age at the time of diagnosis, type of onset (bulbar vs spinal), ALSFRS-R score at diagnosis; King’s stage at diagnosis, and MiToS stage at diagnosis [7, 8]. Pairwise analyses were performed comparing ALS-CN, ALS-INT and ALS-FTD. Partial correlation between UA levels and cognitive tests, controlled for age at the time of testing and educational level, was performed. All tests were two-tailed. Statistical analyses were carried out with the SPSS 29.0 statistical package (SPSS, Chicago, IL, USA).

### Standard protocol approvals, registrations, and patient consents

The study design was approved by the institutional Ethical Committees of the Turin ALS Expert center (Comitato Etico Azienda Ospedaliero-Universitaria Città della Salute e della Scienza, Torino, #0038876). Patients provided written informed consent before enrollment. The database was anonymized according to Italian law for the protection of privacy.

## Results

**Fig. 1:**
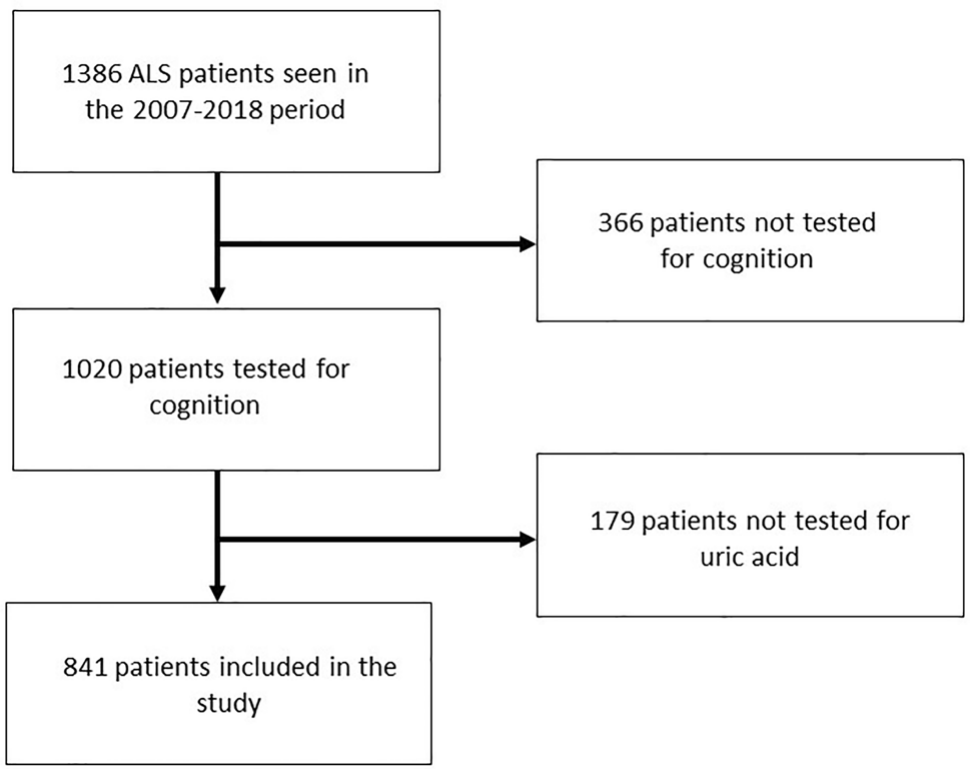
Flow chart reporting the patients’ selection process

A total of 841 ALS patients were included in the study, 422 ALS-CN, 271 ALS-INT and 148 ALS-FTD. The demographic and clinical characteristics of patients are reported in Table 1. A flow chart reporting patients’ selection is reported in Fig. 1.

**Table 1:** Demographic and clinical characteristics of patients included in the study

	Patients *n* = 841	ALS-CN *n* = 422	ALS-INT *n* = 271	ALS-FTD *n* = 148	*p* value
Sex (male)	492 (58.5%)	255 (60.4%)	160 (59.0%)	77 (52%)	*p* = 0.119
Mean age at cognitive examination, years (SD)	66.8 (10.4)	64.6 (10.6)	68.7 (9.5)	69.8 (9.9)	*p* = 0.001
Site of onset (bulbar)	276 (32.8%)	103 (24.4%)	96 (35.4%)	77 (52%)	*p* = 0.001
Mean time from onset to diagnosis, months (SD)	11.3 (11.2)	11.0 (11.8)	11.9 (11.8)	11.0 (8.3)	*p* = 0.598
Mean ALSFRS-R score (SD)	41.1 (5.4)	42.1 (4.6)	41.0 (5.3)	38.9 (7.1)	*p* = 0.001
Mean BMI (SD)	24.7 (4.2)	24.8 (4.1)	25.0 (4.1)	23.9 (4.3)	*p* = 0.03
Mean FVC (SD)	88.1 (26.3)	92.0 (23.0)	88.0 (27.0)	80.2 (30.7)	*p* = 0.001
Familiarity for ALS	95 (11.3%)	40 (9.5%)	23 (8.5%)	32 (21.6%)	*p* = 0.001
King’s staging (1/2/3/4)	393/273/151/25	223/140/48/11	117/91/59/4	53/42/44/9	*p* = 0.001
MiToS staging (0/1/2/3)	590/229/20/2	309/105/7/1	182/83/5/1	99/41/8/0	*p* = 0.058
Mean serum uric acid level (μmol/L)	286.1 (80.9)	288.9 (77.9)	289.7 (74.9)	271.8 (77.3)	*p* = 0.046

The characteristics of each cognitive subgroup are reported. *p* values refer to the comparison between all subgroups and is performed with ANOVA

422 ALS-CN, 271 ALS-INT and 148 ALS-FTD

The mean values of UA were significantly different among the 3 cognitive subgroups of patients, with the lowest values in the ALS-FTD group (ALS-CN, 288.5 μmol/L [SD 78.0]; ALS-INT 289.7 μmol/L [SD 75.5]; ALS-FTD 271.8 μmol/L [74.9]; *p* = 0.046). The frequency of ALS-FTD significantly increased in the 3rd tertile of UA levels (Table 2).

**Table 2:** Frequency of cognitive impairment by tertile of serum UA levels (*p* = 0.046)

	1st tertile (UA levels range 113 to 238 μmol/L)	2nd tertile (UA levels range 238.1 to 303 μmol/L)	3rd tertile (UA levels range 303.1 to 535 μmol/L)
ALS-CN	123 (47.9%)	145 (50.3%)	154 (50.2%)
ALS-INT	77 (30.0%)	89 (30.9%)	105 (35.5%)
ALS-FTD	57 (22.2%)	54 (18.8%)	37 (12.5%)

Binary logistic regression analyses comparing the different cognitive subgroups gave the following results:

- ALS-CN vs ALS-FTD (Table 3): Higher UA levels resulted in being independently associated with FTD (OR 1.32, 95% c.i. 1.01–1.43; *p* = 0.038). As expected, bulbar onset, older age at onset and ALSFRS-R score were also significantly related to the presence of FTD.
- ALS-CN vs ALS-INT (Table 4): UA levels were not correlated with the presence of ALS-INT (OR 1.02, 95% c.i. 0.89–1.15; *p* = 0.82). Only older age at onset results in being correlated with ALS-INT.
- ALS-INT vs ALS-FTD (Table 5): UA levels were independently associated with FTD (OR 1.20, 95% c.i. 1.01–1.49; *p* = 0.047). In this analysis, other factors related to ALS-FTD were bulbar onset and a lower ALSFRS-R score at diagnosis.

**Table 3:** Logistic regression analysis for the comparison between ALS-CN and ALS-FTD. Only significant variables and serum UA are reported

Variables	OR (95% c.i.)	*p* value
ALSFRS-R score (for each point)	1.12 (1.04–1.21)	*p* = 0.001
Age (for each year of age)	1.03 (1.01–1.06)	*p* = 0.005
Site of onset (bulbar)	2.22 (1.20–4.12)	*p* = 0.011
Uric acid (for each decrease of 1 μmol/L)	1.32 (1.01–1.43)	*p* = 0.038

The model included the following variables: ALSFRS-R (continuous), age at the time of diagnosis (continuous), sex (male vs female), site of onset (bulbar vs spinal), education years (continuous), King’s staging (1 vs 2 vs 3 vs 4), MiToS staging (0 vs 1 vs 2 + 3)

**Table 4:** Logistic regression analysis for the comparison between ALS-CN and ALS-INT. Only significant variables and serum UA are reported

Variables	OR (95% c.i.)	*p* value
Age (for each year of age)	1.03 (1.17–1.32)	*p* < 0.0001
Uric acid (for each decrease of 1 μmol/L)	1.02 (0.89–1.15)	*p* = 0.820

The model included the following variables: ALSFRS-R (continuous), age at the time of diagnosis (continuous), sex (male vs female), site of onset (bulbar vs spinal), education years (continuous), King’s staging (1 vs 2 vs 3 vs 4), MiToS staging (0 vs 1 vs 2 + 3)

**Table 5:** Logistic regression analysis for the comparison between ALS-INT and ALS-FTD. Only significant variables and serum UA are reported

Variables	OR (95% c.i.)	*p* value
ALSFRS-R score (for each point)	1.14 (1.06–1.23)	*p* < 0.0001
Uric acid (for each decrease of 1 μmol/L)	1.20 (1.01–1.49)	*p* = 0.047

The model included the following variables: ALSFRS-R (continuous), age at the time of diagnosis (continuous), sex (male vs female), site of onset (bulbar vs spinal), education years (continuous), King’s staging (1 vs 2 vs 3 vs 4), MiToS staging (0 vs 1 vs 2 + 3)

To understand whether UA levels are correlated with specific cognitive domains we performed a binary logistic regression analysis comparing UA levels with the scores of each cognitive test (Table 6). A significant correlation was found between UA serum levels and TMT-B (*p* = 0.033), TMT B-A (*p* = 0.023), BSRT-DR (*p* = 0.025), CPM47 (*p* = 0.003) and ECAS language subscore (*p* = 0.025).

**Table 6:** Partial correlation coefficients for each cognitive and behavioural test

Test	Partial correlation coefficient	*p* value
MMSE	0.061	0.387
FAS	0.044	0.415
CAT	0.161	0.149
FAB	0.017	0.663
Digit Span Forward	0.011	0.77
Digit Span Backward	0.017	0.682
TMT A	– 0.198	0.16
TMT B	– 0.231	**0.033**
TMT B-A	– 0.256	**0.023**
RAVL-IR	– 0.181	0.098
RAVL-DR	– 0.173	0.101
BSRT-IR	0.095	0.063
BSRT-DR	0.087	0.025
ROCF-IR	0.007	0.861
ROCF-DR	0.092	0.478
CPM47	0.271	**0.003**
Clock	0.143	0.281
SET-IA	0.002	0.979
SET-CI	– 0.071	0.347
SET-EA	– 0.033	0.669
SET-GS	– 0.026	0.733
ECAS Language	– 0.224	**0.025**
ECAS Verbal Fluency	– 0.177	0.169
ECAS Executive	– 0.089	0.418
ECAS Memory	0.0381	0.526
ECAS Visuospatial	0.0341	0.546
ECAS ALS specific	– 0.182	0.144
ECAS ALS non-specific	0.093	0.370
ECAS total score	– 0.097	0.400
HADS-A	– 0.121	0.335
HADS-D	– 0.112	0.802
FrSBe apathy	– 0.051	0.905
FrSBe disinhibition	0.058	0.362
FrSBe dysexecutive	0.019	0.656
FrSBe total score	0.035	0.714

*BSRT* Babcock Story Recall Test, immediate (IR) and delayed recall (DR); *CAT* category fluency test, *Clock* Clock Drawing Test, *CPM47* Raven’s Colored Progressive Matrices, *ECAS* Edinburgh Cognitive and Behavioural ALS Screen, *FAB* Frontal Assessment Battery, *FAS* letter fluency test, *FrSBe* Frontal Systems Behavior Scale, *HADS-A* Hospital Anxiety and Depression Scale – Anxiety, *HADS-D* Hospital Anxiety and Depression Scale – Depression, *MMSE* Mini-Mental State Examination, *RAVL* Rey Auditory Verbal Learning Test, immediate (IR) and delayed recall (DR); *ROCF* Rey-Osterrieth Complex Figure Test, immediate recall (IR) and delayed recall (DR); *SET-EA* Story-Based Empathy Task—Emotion Attribution, *SET-IA* Story-Based Empathy Task—Intention Attribution, *SET-CI* Story-Based Empathy Task—Causal Inference, *SET-GS* Story-Based Empathy Task – Global Score, *TMT* Trail-Making Test A, B, and B–A

An exploratory analysis was also performed on the 61 patients with *C9orf72* GGGGCC repeat expansion. Of these patients, 21 were CN-ALS, 14 ALS-INT and 26 ALS-FTD. UA mean levels were not different in the 3 cognitive groups, although UA levels in ALS-FTD were lower than in the other two groups (CN-ALS 277.2 μmol/L [SD 88.0], ALS-INT 276.0 μmol/L [SD 60.1], ALS-FTD 263.5 μmol/L [78.5], *p* = 0.82), although they were lower in the ALS-FTD group. In accordance, all binary logistic regression analyses did not find UA as a significant predictor of cognitive impairment.

## Discussion

In this large cohort of ALS patients evaluated with an extensive battery for cognitive impairment at the time of diagnosis we found that lower UA levels were independently correlated with the occurrence of FTD. Conversely, when analyzing the *C9orf72* patients, this correlation was not found, although FTD subjects had slightly lower UA levels. Besides, we found that lower serum UA levels were significantly correlated with impairment in tests assessing Executive Functions (TMT B-A), Verbal Memory (BSRT Delayed Recall), Cognitive Flexibility (TMT B), Non-Verbal Intelligence (CPM47) and Language (ECAS Language subdomain).

High serum UA levels have been associated with slower progression of ALS [9], although with an effect primarily limited to female [10]. A bidirectional Mendelian randomization (MR) study did not support a significant contribution of UA to the risk of ALS [11], but the relationship of UA with ALS prognosis and/or cognitive involvement was not explored. On the other side, a serum metabolome analysis, based on a two-sample MR, reported a significant protective link between high serum UA levels and disease risk in ALS but not in Parkinson’s disease (PD) [12]. Therefore, at least in the case of ALS, a reverse causality cannot be definitely excluded.

On the basis of the evidence, pharmacological trials have been undertaken both in PD and in ALS to explore the possible effect of inosine, a drug that acts to increase the level of serum and cerebrospinal fluid UA [13, 14]. Despite a significant target engagement (serum urate increase), no clinical effect on disease progression was detected. Of note, cognition was not explored in the trial on ALS patients [14].

To the best of our knowledge, the only study that has explored the role of serum UA serum levels on cognitive impairment in a small ALS cohort reported that patients cognitively impaired showed significantly lower levels of UA [15]. In that study, UA levels were significantly correlated with the Language and Executive Function subdomains of ECAS (*p* < 0.01 and *p* = 0.047 respectively). In our cohort, only ECAS Language subdomain was significantly related to UA serum levels, but in addition our extensive cognitive series of tests detected low UA levels in other cognitive domains, i.e., executive functions (TMT B-A), verbal memory (BSRT delayed recall), cognitive flexibility (TMT B), and non-verbal intelligence (CPM47).

In our cohort, patients with ALS-INT had levels of UA similar to ALS-CN, but significantly higher than those with ALS-FTD. This finding could indicate that higher UA levels at diagnosis can be protective in patients with mild cognitive impairment, preventing their progression to full-blown FTD. However, to confirm our hypothesis longitudinal studies are necessary.

In an exploratory analysis, we did not find any correlation of serum UA levels and FTD in *C9orf72* patients, despite their lower levels of serum UA. Such discrepancy could be due to the relatively small number of *C9orf72* patients. However, we can also hypothesize that the strong direct effect of this gene on the occurrence of FTD overcome the protective effect of UA on cognitive function [16]. Studies on larger series of *C9orf72* patients will help to better understand this aspect.

UA has different, two-faced, mechanisms related to neuroprotection/neurotoxicity in general. Its protective effects could be related to an important anti-oxidative activity in human plasma reducing the formation of peroxynitrite [17, 18], and an anti-inflammatory activity; in fact, UA is thought to prevent the disruption of blood–brain barrier integrity and to increase IL-6 levels, with the subsequent activation of the IL-6/signal transducer and of transcription 3 (STAT3) signaling pathway [19, 20]. However, UA may also have pro-oxidative activity through lipid peroxidation, free radical formation [21], and pro-inflammatory activity by triggering interleukin (IL)-1b-mediated inflammation via activation of the nucleotide-binding and oligomerization domain (NOD)-like receptor protein (NLRP) 3 inflammasome, and also through the nuclear factor kappa-light chain-enhancer of activated B cells (NF-kB) signaling pathway [22, 23]. This two-faced effect of UA on oxidative and inflammatory processes reflects on cognitive function. In fact, high UA levels have been shown to be related to worse cognitive function in subjects with cardiovascular cerebrovascular and chronic kidney disorders, including elderly hemodialysis subjects [3]. Besides, in subjects with high vascular burden low UA levels were associated with poorer cognitive performances, manifested by lower global cognitive, memory, executive, and visuospatial scores [24]. On the contrary, high UA levels were found to be related to better cognitive function in subjects older than 60 [25–28]. In addition, higher UA levels are reported to be protective against cognitive damage in PD [29], while their effect on Alzheimer disease are inconclusive [30]. In summary, it appears that UA can be detrimental to cognitive function in subjects with co-morbid systemic disorders, while it can be protective in patients with neurodegenerative disorders and also in healthy elderly.

This study is not without limitations. First, cognitive status and serum UA levels have been evaluated at the time of diagnosis and, therefore, represents the condition at an early-intermediate stage of the disease. Longitudinal data assessing UA levels and cognitive conditions would be important to better understand the persistence of a protective role of UA, in particular in patients converting from normal cognition to cognitive impairment. However, in our series the effect of UA was independent of clinical staging, which has been shown to be strongly related to the severity of cognitive impairment [31, 32]. Second, ECAS was performed only in patients seen from 2016, when the validated Italian translation became available.

In this large cohort of ALS patients with extensive cognitive and behavioural evaluation performed at the time of diagnosis we found that high UA serum levels are correlated with a reduced frequency of co-morbid FTD. Patients with intermediate cognitive impairment (ALSci, ALSbi and ALScbi) showed UA levels similar to those with ALS-CN but higher than those of ALS-FTD, a possible indication that higher UA levels can prevent or delay the deterioration of cognitive function. However, UA serum levels were not significantly correlated with the presence of FTD in patients carrying *C9orf72* repeat expansion. Together with the proposed role of UA as a prognostic factor in ALS, our data show that UA can also be a protective factor for the cognitive-behavioural component of the disease. Further study on UA will better elucidate the mechanisms through which this metabolite may exert its protective activity in ALS.

### Supplementary Information

Below is the link to the electronic supplementary material.

Supplementary file1 (DOCX 14 KB)

Supplementary file2 (DOCX 33 KB)

## Data Availability

Data will be available upon reasonable request by interested researchers.
