# Supplementary material for: High serum uric acid levels are protective against cognitive impairment in amyotrophic lateral sclerosis
Source: J Neurol. 2023 Oct 25;271(2):955–61. doi: 10.1007/s00415-023-12056-8 (PMC10827906; doi:10.1007/s00415-023-12056-8)
Supplement: Supplementary file 1 — Supplementary file1 (DOCX 14 KB) [file 415_2023_12056_MOESM1_ESM.docx]

**Supplemental material**

**Additional methods**

The following neuropsychological battery was used: Letter Fluency test (FAS); Category Fluency Test (CAT); Frontal Assessment Battery (FAB); Trail Making Test (TMT) A, B and B-A; Rey-Osterrieth Complex Figure Test (ROCF), immediate (IR) and differed recall (DR); Rey Auditory Verbal Learning Test (RAVL), immediate (IR) and differed recall (DR); Babcock Story Recall Test (BSRT), immediate (IR) and differed recall (DR); Digit Span Forward and Backward; Clock Drawing Test; Raven’s Colored Progressive Matrices (CPM47); Mini Mental State Examination (MMSE). From 2016, patients also underwent the Edinburgh Cognitive and Behavioural ALS Screen (ECAS) using the published Italian version (Poletti et al, 2018). After 2018 patients were also tested with Story-based Empathy Task (SET) evaluating cognitive and affective Theory of Mind. The most recent Italian normative were used to correct for age, sex, and education the raw scores of tests.

Neurobehavioral dysfunction was determined with the Frontal Systems Behaviour Scale (FrSBe), using the Family-form evaluated by a close relative/caregiver (scores: normal ≤59, borderline 60-64; pathological ≥65). For the purpose of this study, we considered the change in points for each of the 3 domains of FrSBe (apathy, disinhibition, executive) from the before disease to the disease scores. If a subject had scores reflecting a frontal systems abnormality both in the premorbid and in the post-illness forms, he/she was considered pathological only if there was an increase of ≥10 points at the T-score between the two forms (Montuschi et al, 2015). Anxiety and depression were assessed with the Hospital Anxiety and Depression Scale (HADS); the item “I feel slowed down” was discussed with patients in order to have him/her not to refer to physical disability (Montuschi et al, 2015).

**Supplementary references**

Montuschi A, Iazzolino B, Calvo A, Moglia C, Lopiano L, Restagno G, Brunetti M, Ossola I, Lo Presti A, Cammarosano S, Canosa A, Chiò A. Cognitive correlates in amyotrophic lateral sclerosis: a population-based study in Italy. J Neurol Neurosurg Psychiatry. 2015 Feb;86(2):168-73. doi: 10.1136/jnnp-2013-307223. PMID: 24769471.

Poletti B, Solca F, Carelli L, Faini A, Madotto F, Lafronza A, Monti A, Zago S, Ciammola A, Ratti A, Ticozzi N, Abrahams S, Silani V. Cognitive-behavioral longitudinal assessment in ALS: the Italian Edinburgh Cognitive and Behavioral ALS screen (ECAS). Amyotroph Lateral Scler Frontotemporal Degener. 2018 Aug;19(5-6):387-395. doi: 10.1080/21678421.2018.1473443. PMID: 29804470.
